# Disruption of mitochondrial electron transport impairs urinary concentration via AMPK-dependent suppression of aquaporin 2

**DOI:** 10.1172/jci.insight.182087

**Published:** 2024-11-22

**Authors:** Joshua S. Carty, Ryoichi Bessho, Yvonne Zuchowski, Jonathan B. Trapani, Olena Davidoff, Hanako Kobayashi, Joseph T. Roland, Jason A. Watts, Andrew S. Terker, Fabian Bock, Juan Pablo Arroyo, Volker H. Haase

**Affiliations:** 1 Division of Nephrology and Hypertension, Department of Medicine, Vanderbilt University Medical Center and Vanderbilt University School of Medicine, Nashville, Tennessee, USA.; 2 Research and Medical Services, Department of Veterans Affairs, Tennessee Valley Healthcare System, Nashville, Tennessee, USA; 3 Section of Surgical Sciences, Epithelial Biology Center, Vanderbilt University Medical Center, Nashville, Tennessee, USA.; 4 Epigenetics and Stem Cell Laboratory, National Institute of Environmental Health Sciences, NIH, Research Triangle Park, North Carolina, USA.; 5 Department of Molecular Physiology and Biophysics, Vanderbilt University School of Medicine, Nashville, Tennessee, USA.

**Keywords:** Cell biology, Nephrology, Bioenergetics, Epithelial transport of ions and water, Mitochondria

## Abstract

Urinary concentration is an energy-dependent process that minimizes body water loss by increasing aquaporin 2 (AQP2) expression in collecting duct (CD) principal cells. To investigate the role of mitochondrial (mt) ATP production in renal water clearance, we disrupted mt electron transport in CD cells by targeting ubiquinone (Q) binding protein QPC (UQCRQ), a subunit of mt complex III essential for oxidative phosphorylation. QPC-deficient mice produced less concentrated urine than controls, both at baseline and after type 2 vasopressin receptor stimulation with desmopressin. Impaired urinary concentration in QPC-deficient mice was associated with reduced total AQP2 protein levels in CD tubules, while AQP2 phosphorylation and membrane trafficking remained unaffected. In cultured inner medullary CD cells treated with mt complex III inhibitor antimycin A, the reduction in AQP2 abundance was associated with activation of 5′ adenosine monophosphate–activated protein kinase (AMPK) and was reversed by treatment with AMPK inhibitor SBI-0206965. In summary, our studies demonstrated that the physiological regulation of AQP2 abundance in principal CD cells was dependent on mt electron transport. Furthermore, our data suggested that oxidative phosphorylation in CD cells was dispensable for maintaining water homeostasis under baseline conditions, but necessary for maximal stimulation of AQP2 expression and urinary concentration.

## Abstract 2

This manuscript demonstrates that disruption of mitochondrial electron transport in collecting duct epithelial cells impairs the kidney’s ability to concentrate urine by dysregulating aquaporin-2 expression.

## Graphical abstract

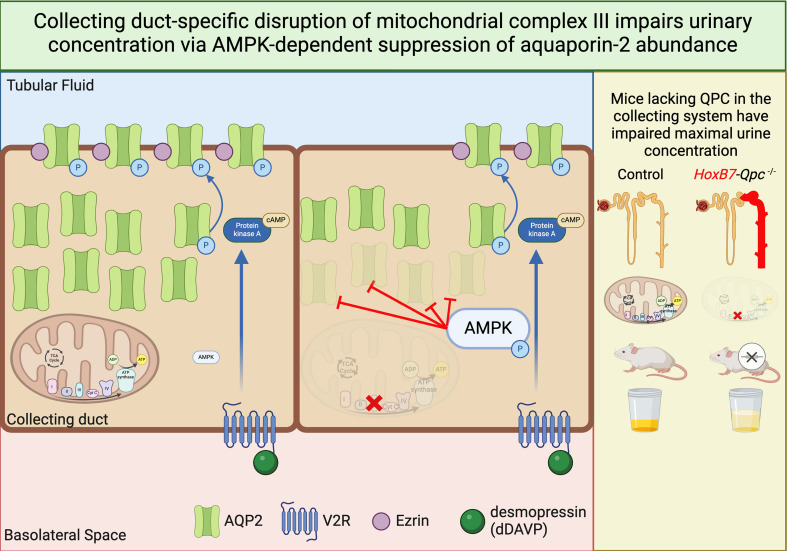

## Introduction

In the kidney, epithelial transport of ions, solutes, and water are energy intensive processes that require adenosine 5′-triphosphate (ATP). Key biochemical pathways involved in ATP generation are the tricarboxylic acid (TCA) cycle, oxidative phosphorylation, which is oxygen-dependent and coupled to mitochondrial (mt) electron transport, and glycolysis, which does not require oxygen (1). Oxidation of 1 glucose molecule generates approximately 30 to 38 molecules of ATP through the combination of these processes. In the kidney, ATP requirements vary along the nephron and correlate with mitochondrial density (2, 3). In mitochondria, the electron transport chain (ETC) is composed of 4 large multisubunit enzymatic complexes that utilize high-energy electrons generated from the oxidation of NADH and FADH_2_ to pump protons into the intermembrane space, thus creating a proton chemiosmotic gradient that fuels ATP generation via ATP synthase (4, 5). Mt complex I (NADH:ubiquinone oxidoreductase) and complex II (succinate:ubiquinone oxidoreductase) oxidize NADH and FADH_2_, respectively, whereas complex III (coenzyme Q:cytochrome *c* oxidoreductase) transfers high-energy electrons from ubiquinol to cytochrome *c* (6). Complex IV (cytochrome *c* oxidase) then uses cytochrome *c* for the reduction of molecular oxygen to H_2_O. Less efficient ATP production is achieved through glycolysis, a biochemical process that does not require oxygen and contributes to cellular ATP generation in low O_2_ environments, such as the renal medulla (7, 8).

To achieve urinary concentration, collecting duct (CD) cells, in concert with other cell types, move solutes against an osmotic gradient to create a hypertonic milieu in the medullary interstitium, which requires ATP (9). The hypertonic medullary interstitium then favors the movement of water from the lumen of the CD into the medullary interstitium and subsequently into peritubular capillaries (9). Water permeability in the CD is regulated by the apical presence or absence of water channel aquaporin 2 (AQP2). When water conservation is required, apical membrane AQP2 expression becomes more abundant, thereby increasing water permeability and water reabsorption into interstitium and peritubular capillaries (10, 11). This process requires signaling through the type 2 vasopressin receptor (V2R), which is expressed in the distal nephron. In collecting ducts, V2R is stimulated by the antidiuretic hormone arginine vasopressin (AVP), resulting in increased expression, phosphorylation, and membrane abundance of AQP2.

To what degree oxidative phosphorylation is required for urinary concentration and AQP2 function is unclear and has not been investigated directly. To address this question, we disrupted mt electron transport in CD principal cells by targeting ubiquinol–cytochrome *c* reductase UQCRQ, a subunit of mt complex III, also known as ubiquinone (Q) binding protein, QPC. For these studies, we took advantage of a *Cre*-recombinase transgene under control of the homeobox B7 (*HoxB7*) promoter (12). Inactivation of QPC disrupts mt electron transport, resulting in the inhibition of oxidative phosphorylation and mt ATP production (6). We found that mice with CD-specific inactivation of QPC were not able to maximally concentrate urine due to impaired induction of AQP2 expression in principal cells, which was associated with 5′ adenosine monophosphate–activated protein kinase (AMPK) activation. Inhibition of mt complex III in cultured CD cells resulted in a reduction of AQP2 expression that was reversed by pharmacological AMPK inhibition. Taken together, our data establish that mt electron transport is required for maximal urinary concentration and regulates kidney water reabsorption in an AMPK-dependent manner.

## Results

### Efficient QPC deletion in HoxB7-Qpc^–/–^ mice.

**Figure 1:**
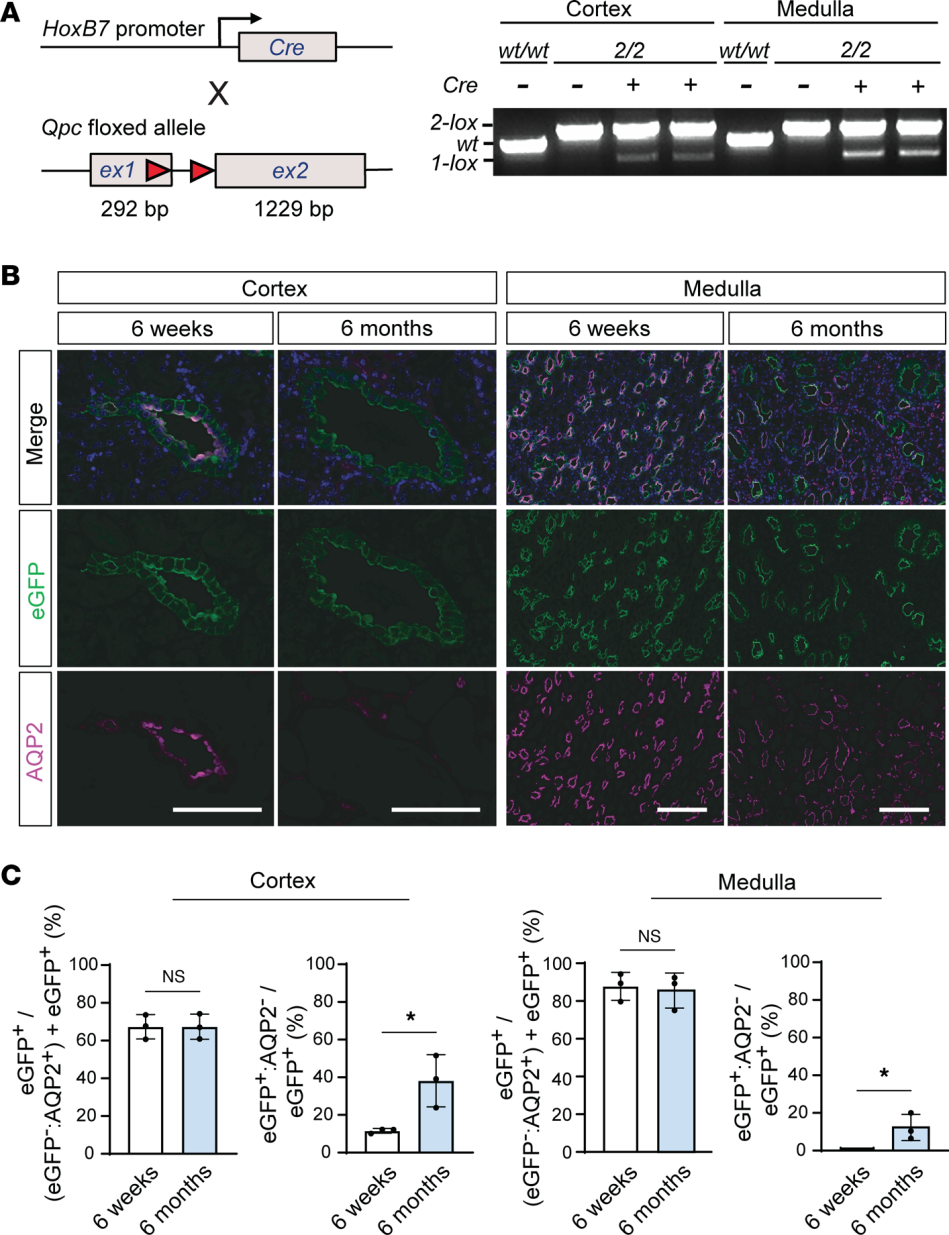
Efficient recombination of *Qpc* in *HoxB7-Qpc^–/–^* kidneys. (**A**) Schematic illustrating the location of targeted DNA sequences within the conditional *Qpc* allele; *loxP* sites are depicted by red arrows. The sizes of the *Qpc* exons (ex) 1 and 2 are shown in base pairs (bp). PCR analysis of total genomic DNA isolated from kidney cortex and medulla of 12-week-old wild-type (wt), *HoxB7-Qpc^−/−^* mutants, and *Cre^–^* littermate control mice. The genotype of mice is indicated by 2, representing the nonrecombined 2-lox allele and by 1, representing the recombined 1-lox allele; + or – indicates the presence or absence of the *Cre* transgene. (**B**) Representative images of immunofluorescent (IF) staining for aquaporin 2 (AQP2) and enhanced green fluorescent protein (eGFP) in formalin-fixed, paraffin-embedded kidney sections. Kidney tissues were obtained from 6-week- and 6-month-old *HoxB7-mT/mG-Qpc^−/−^* mice. (**C**) Quantification of eGFP^+^ and eGFP^+^AQP2^–^ tubules in kidney cortex and medulla expressed as percentage of (eGFP^–^AQP2^+^ + eGFP^+^) or total number of eGFP^+^ tubules, respectively; *n* =3, each. Percentages are represented as average mean values ± SD. *P < 0.05 by 2-tailed Student’s *t* test. NS, not significant. Scale bars: 100 μm. *HoxB7*, homeobox B7; *Qpc*, gene encoding ubiquinone-binding protein QPC.

For the inactivation of QPC in the renal collecting system, *HoxB7-cre* transgenics were bred with mice carrying the conditional *Uqcrq* (*Qpc*) 2-lox allele, generating *HoxB7-cre^tg/+^* *Qpc^fl/fl^* mice, referred to as *HoxB7-Qpc^–/–^* mutants (13, 14). The *HoxB7-cre* transgene induces robust Cre-mediated recombination in the entire ureteric bud (UB) epithelium, resulting in efficient gene targeting in the adult collecting system (13). To identify cells that have undergone Cre-mediated recombination, we took advantage of the *ROSA26-ACTB-tdTomato-EGFP* double-fluorescent Cre recombinase reporter allele, from here on referred to as *mT/mG* (15). Genomic DNA analysis by PCR and immunofluorescent (IF) staining for membrane-bound enhanced green fluorescence protein (eGFP) demonstrated efficient recombination in CD cells (Figure 1). Comparable degrees of *Qpc* recombination were found in kidneys from 6-week- and 6-month-old mice, suggesting that the population of *Qpc^–/–^* CD cells remained stable in *HoxB7-Qpc^–/–^* mutants (Figure 1, B and C). Furthermore, we noted that CD tubules were mildly dilated in *HoxB7-Qpc^–/–^* mutants (Figure 1B).

### AQP2-expression in CD epithelium is reduced in HoxB7-Qpc^–/–^ mice.

eGFP^+^ cells that coexpress AQP2 (eGFP^+^AQP2^+^) in *HoxB7-mT/mG-Qpc^–/–^*mutant mice represent principal cells within CD tubules. Given the high efficiency of *HoxB7-cre*, cortical AQP2^+^ tubules not expressing eGFP (eGFP^–^AQP2^+^) most likely represented connecting tubules, which have been shown to be derived from renal vesicles and not from the UB (16, 17). However, some of the cortical eGFP^–^AQP2^+^ cells were tdTomato^–^, indicating that the *mT/mG* reporter was not active (Supplemental Figure 1; supplemental material available online with this article; https://doi.org/10.1172/jci.insight.182087DS1). In the medulla, a few eGFP^–^AQP2^+^ cells were found that either represented cells without a history of Cre expression or cells that did not express the *mT/mG* reporter allele (Supplemental Figure 1). Unexpectedly, we found that a relatively high proportion of eGFP^+^ tubules did not express AQP2 (eGFP^+^AQP2^–^) in 6-month-old *HoxB7-mT/mG-Qpc^–/–^* mutants, raising the possibility that mt electron transport might be involved in the regulation of AQP2 in CD epithelium (Figure 1C).

**Figure 2:**
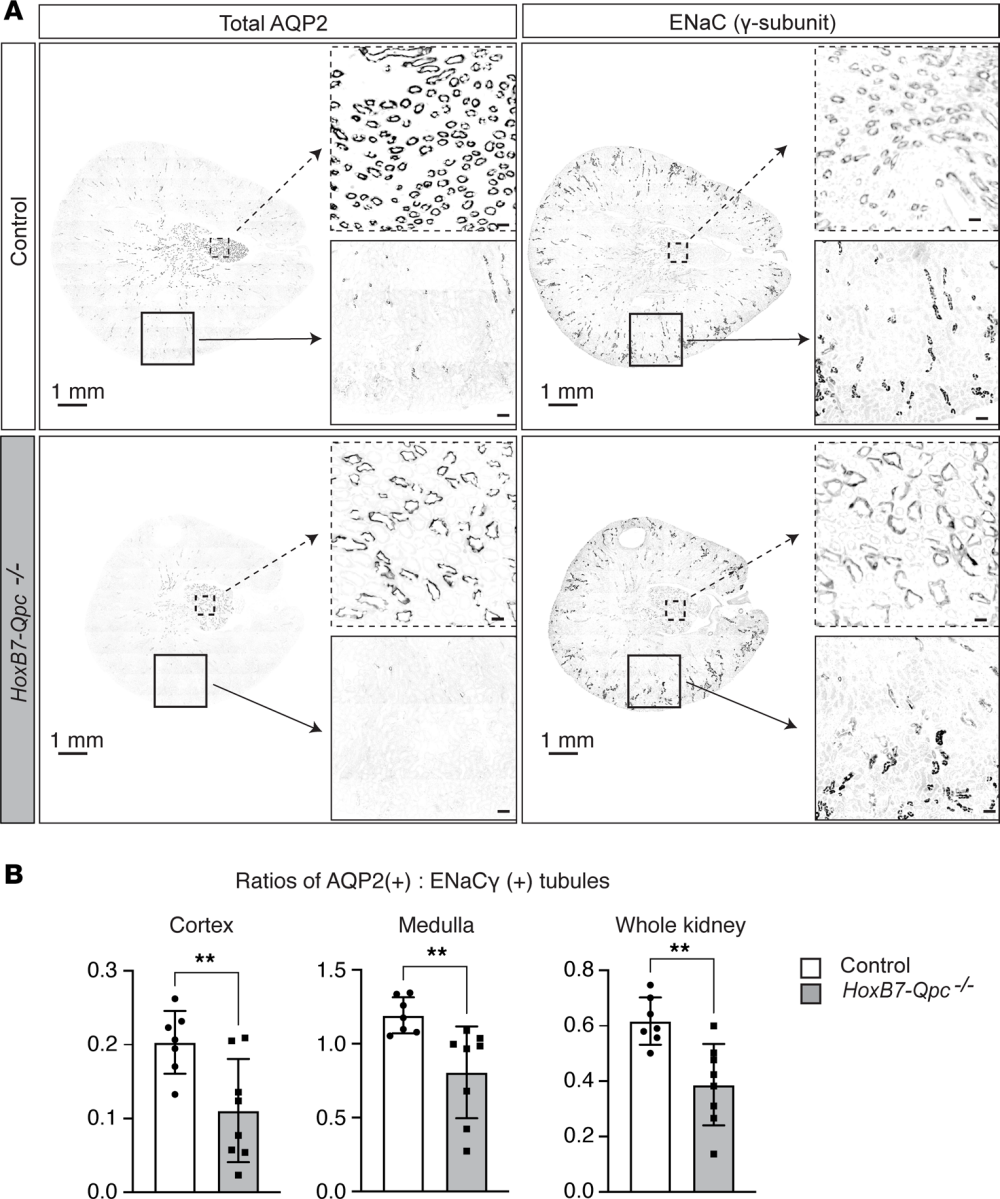
AQP2-expressing tubules are reduced in *HoxB7-Qpc^–/–^* kidneys. (**A**) Representative images of immunofluorescent staining for AQP2 and ENaC γ-subunit of whole horizontal kidney sections from *Cre^–^* littermate control and *HoxB7-Qpc^–/–^* mice. Low- and high-power magnifications of representative tissue areas are shown by dashed boxes and unbroken line boxes, respectively. (**B**) Ratios of AQP2^+^ to ENaCγ^+^ tubules in cortex, medulla, or entire kidney cross section. For each kidney section, all tubules were counted: *Cre^–^* littermate control (*n* = 7) and *HoxB7-Qpc^–/–^* mutants (*n* = 8). The ratio of AQP2^+^ to ENaCγ^+^ tubules was decreased in *HoxB7-Qpc^–/–^* mutants compared with controls. Data are represented as average mean values ± SD. **P < 0.01 by 2-tailed Student’s *t* test. Scale bars: 1 mm (whole kidney sections), 30 μm (low-power magnification), and 100 μm (high-power magnification). AQP2, aquaporin 2; ENaC, epithelial sodium channel.

To investigate whether a reduction in kidney CD cell mass could account for the reduced presence of eGFP^+^AQP2^+^ cells in *HoxB7-Qpc^–/–^* mice, we used IF staining to quantify AQP2^+^ tubules and tubules that expressed the γ-subunit of the epithelial sodium channel (ENaCγ), a key marker of principal cells. We found that relative to controls, the AQP2-expressing tubules were reduced, which was most pronounced in the cortex (Figure 2 and Supplemental Figure 2). In contrast, there was no difference in the number of ENaCγ^+^ tubules between control and *HoxB7-Qpc^–/–^* mutants. Next, we calculated the ratios of AQP2^+^ to ENaCγ^+^ tubules for *HoxB7-Qpc^–/–^* mutant *Cre^–^* littermate control mice. The ratios of AQP2^+^ to ENaCγ^+^ tubules were reduced in both kidney cortex and medulla of kidneys from *HoxB7-Qpc^–/–^* mutants compared with controls (Figure 2). Taken together, our data suggested that disruption of mt electron transport in *HoxB7-Qpc^–/–^* mice decreases the expression of AQP2 in CD epithelial cells relative to total CD cell mass.

### Mt electron transport maintains urinary concentrating ability by regulating AQP2 abundance in CD epithelium.

**Figure 3:**
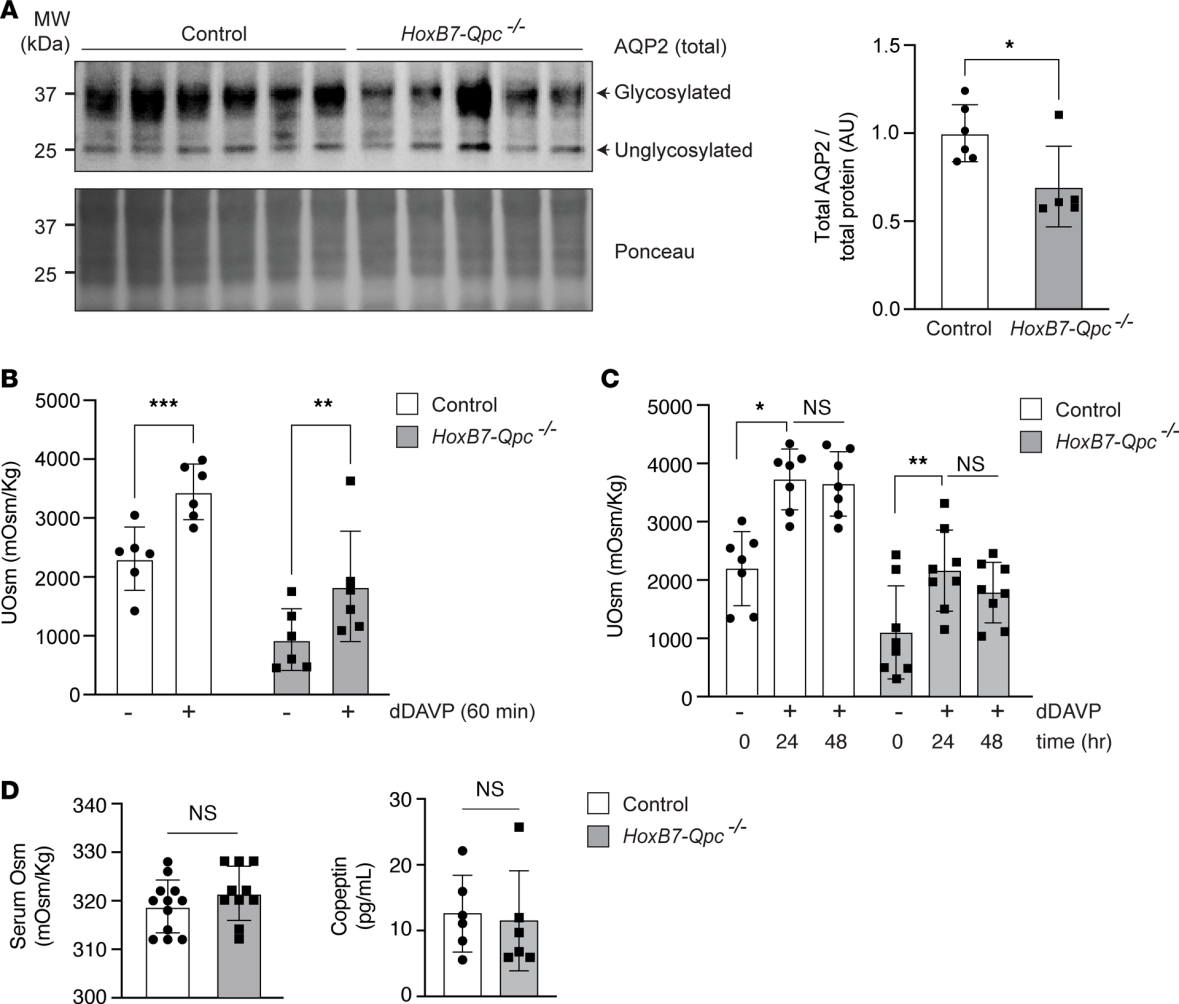
Total AQP2 protein abundance and urine osmolality are decreased in *HoxB7-Qpc^–/–^* mice. (**A**) Total aquaporin 2 (AQP2) in whole-kidney lysates from *Cre^–^* littermate control and *HoxB7-Qpc^–/–^* mice. (**B**) Urine osmolality (UOsm) at baseline and following acute desmopressin (dDAVP) challenge in *Cre^–^* littermate control and *HoxB7-Qpc^–/–^* mice; *n* = 6, each. (**C**) UOsm at baseline and after repeated dDAVP challenge in *Cre^–^* littermate control and *HoxB7-Qpc^–/–^* mice; *n* = 7 and 8, respectively. (**D**) Serum Osm and copeptin levels at baseline in *Cre^–^* littermate control and *HoxB7-Qpc^–/–^* mice; *n* = 12 and 10, respectively. Data are represented by mean values ± SD. *P < 0.05; **P < 0.01; ***P < 0.001 by 2-tailed Student’s *t* test (**A** and **D**) or 2-way ANOVA followed by Šidák’s multiple-comparison test (**B** and **C**). NS, not significant. The interaction between genotype and treatment was not significant in **B** or **C**. AU, arbitrary units.

AQP2 is required to reabsorb water from the collecting duct lumen, and lack of AQP2 expression in CD cells results in dilute urine and increased water excretion. To investigate whether AQP2 expression was affected by QPC inactivation, we assessed total cellular AQP2 levels by immunoblotting. We found that total AQP2 protein was decreased in whole-kidney lysates from *HoxB7-Qpc^–/–^* mice, which is consistent with the decrease in the number of AQP2^+^ tubules (Figure 3A). Aquaporin 3 (AQP3) is also expressed in the CDs and is involved in water transport (18). To assess the effects of QPC deletion on AQP3 expression, we performed immunoblotting. Although there was a trend toward decreased expression of AQP3, we found no statistically significant difference between *HoxB7-Qpc^–/–^* mutants and *Cre^–^* littermate controls (Supplemental Figure 3). Because AQP2 expression was reduced in *HoxB7-Qpc^–/–^* mutants, we next examined to what degree urinary concentrating ability in mutant mice was affected by disruption of mt electron transport in CD cells (Figure 3B). We found that urine from 10- to 12-week-old *HoxB7-Qpc^–/–^* mice was less concentrated compared with *Cre^–^* littermate controls at baseline (936 ± 526 vs. 2311 ± 535 mOsm/kg) (Figure 3B) and after 12-hour water restriction (2568 ± 108 vs. 3517 ± 213 mOsm/kg, *n* = 3, each; 2-sided Student’s *t* test, *P* < 0.01). When stimulated with the V2R-specific agonist desmopressin (dDAVP), *HoxB7-Qpc^–/–^* urinary concentration increased; however, the maximal urinary concentration was blunted relative to controls acutely (1837 ± 939 vs. 3447 ± 474 mOsm/kg) (Figure 3B) and chronically (2149 ± 696 vs. 3710 ± 520.9 mOsm/kg at 24 hours and 1771 ± 520 vs. 3633 ± 553.5 mOsm/kg at 48 hours) (Figure 3C). To evaluate whether the blunted urinary concentration affected baseline water homeostasis, we measured serum osmolality and copeptin (a surrogate for AVP) levels and found no differences between control or *HoxB7-Qpc^–/–^* mice (318.8 ± 5.4 vs. 321.4 ± 5.5 mOsm/kg and 12.58 ± 5.8 vs. 11.48 ± 7.6 pg/mL, respectively) (Figure 3D). Moreover, blood electrolytes were not different between controls and *HoxB7-Qpc^–/–^* mutants (Supplemental Table 2). Together, these data suggest that *HoxB7-Qpc^–/–^* mice developed an impaired ability to concentrate urine, but were still able to maintain appropriate serum osmolality and blood electrolyte levels at baseline.

**Figure 4:**
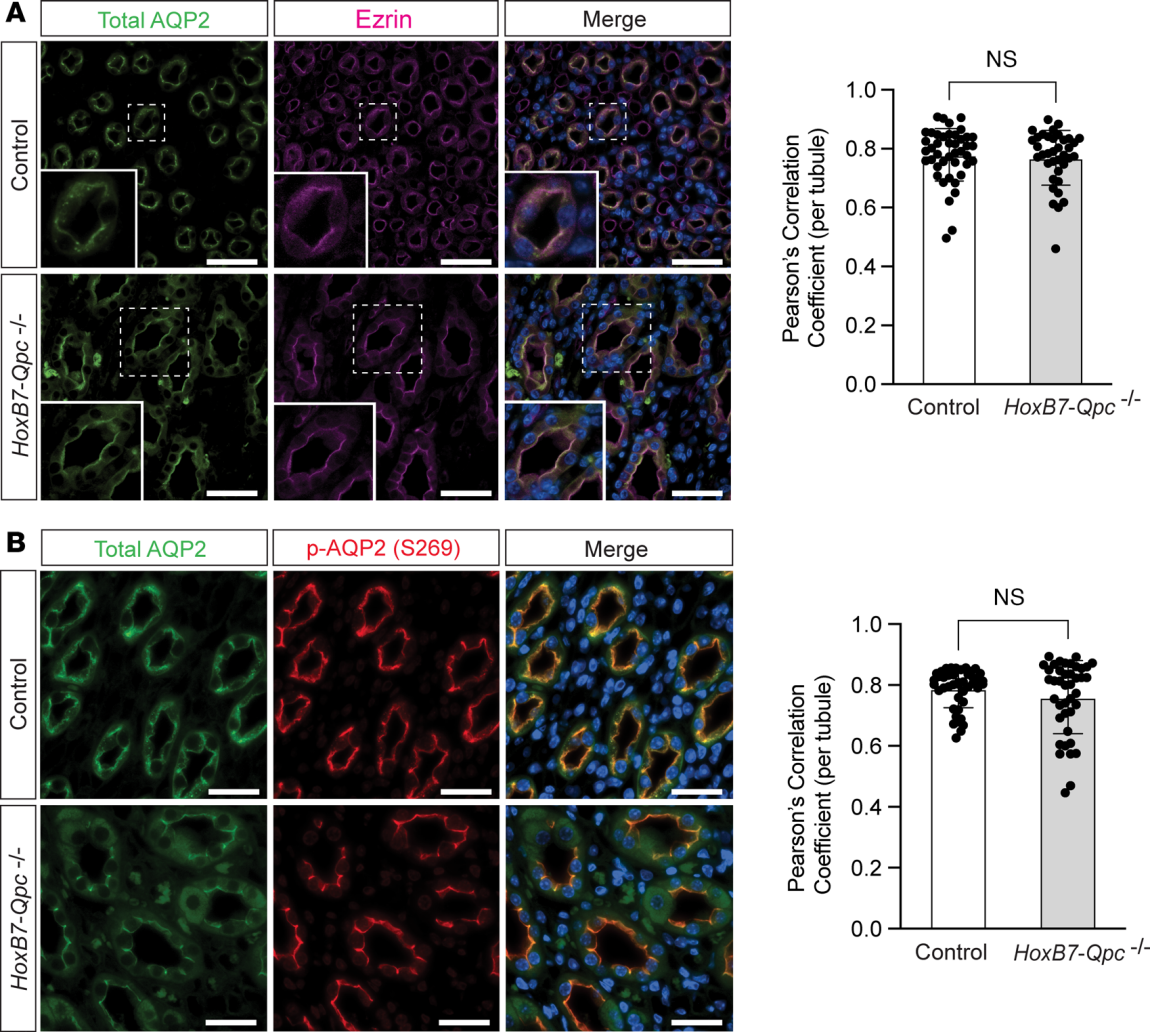
AQP2 phosphorylation and membrane trafficking in *HoxB7-Qpc^–/–^* kidneys after dDAVP stimulation are comparable to controls. (**A**) Representative immunofluorescent (IF) costaining for total AQP2 (green) and ezrin (magenta) and (**B**) IF costaining for total AQP2 (green) and p-AQP2 (S269) (red) in control and *HoxB7-Qpc^–/–^* kidneys following treatment of control and *HoxB7-Qpc^–/–^* mice with desmopressin (dDAVP). Graphs show Pearson’s correlation between relative normalized IF intensities per tubule for total AQP2 and ezrin IF staining intensity (**A**) or total AQP2 and p-AQP2 (S269) IF staining intensity (**B**) for *Cre^–^* littermate control and *HoxB7-Qpc^–/–^* kidneys, respectively; *n* = 4 mice per group, 10 tubules per mouse. Nuclei (blue) were stained with DAPI (4′,6-diamidino-2-phenylindole). Data are represented as mean Pearson’s correlation coefficient per tubule ± SD and were evaluated with a 2-tailed Student’s *t* test. NS, not significant. Scale bars: 40 μm. AQP2, aquaporin 2.

To evaluate whether QPC inactivation interfered with V2R-mediated membrane expression and/or phosphorylation of AQP2 in *HoxB7-Qpc^–/–^* mice, we used IF staining to assess whether AQP2 colocalized with apical membrane protein ezrin and phosphorylated AQP2 (p-AQP2 [S269]) following dDAVP treatment. Ezrin, an actin binding protein that belongs to the ezrin/radixin/moesin family, directly interacts with AQP2 and colocalizes with AQP2 to the apical membrane in response to dDAVP (19). We found no difference in membrane colocalization between ezrin and AQP2, suggesting that QPC inactivation did not interfere with the ezrin-AQP2 interaction and its trafficking to the apical membrane of CD cells (Figure 4A). Furthermore, we found that staining for total AQP2 colocalized with p-AQP2 (S269) in both, control and *HoxB7-Qpc^–/–^* mice, suggesting that phosphorylation of AQP2 (S269) was preserved in *HoxB7-Qpc^–/–^* mutants (Figure 4B).

Taken together, these data demonstrate that mt electron transport is required to achieve maximal urinary concentration and that disruption of mt electron transport impairs urinary concentrating ability due to a reduction in the cellular abundance of AQP2, but not in AQP2 phosphorylation or membrane trafficking, in response to dDAVP.

### AMPK regulates AQP2 in CD cells with mt electron transport disruption in vitro and in vivo.

**Figure 5:**
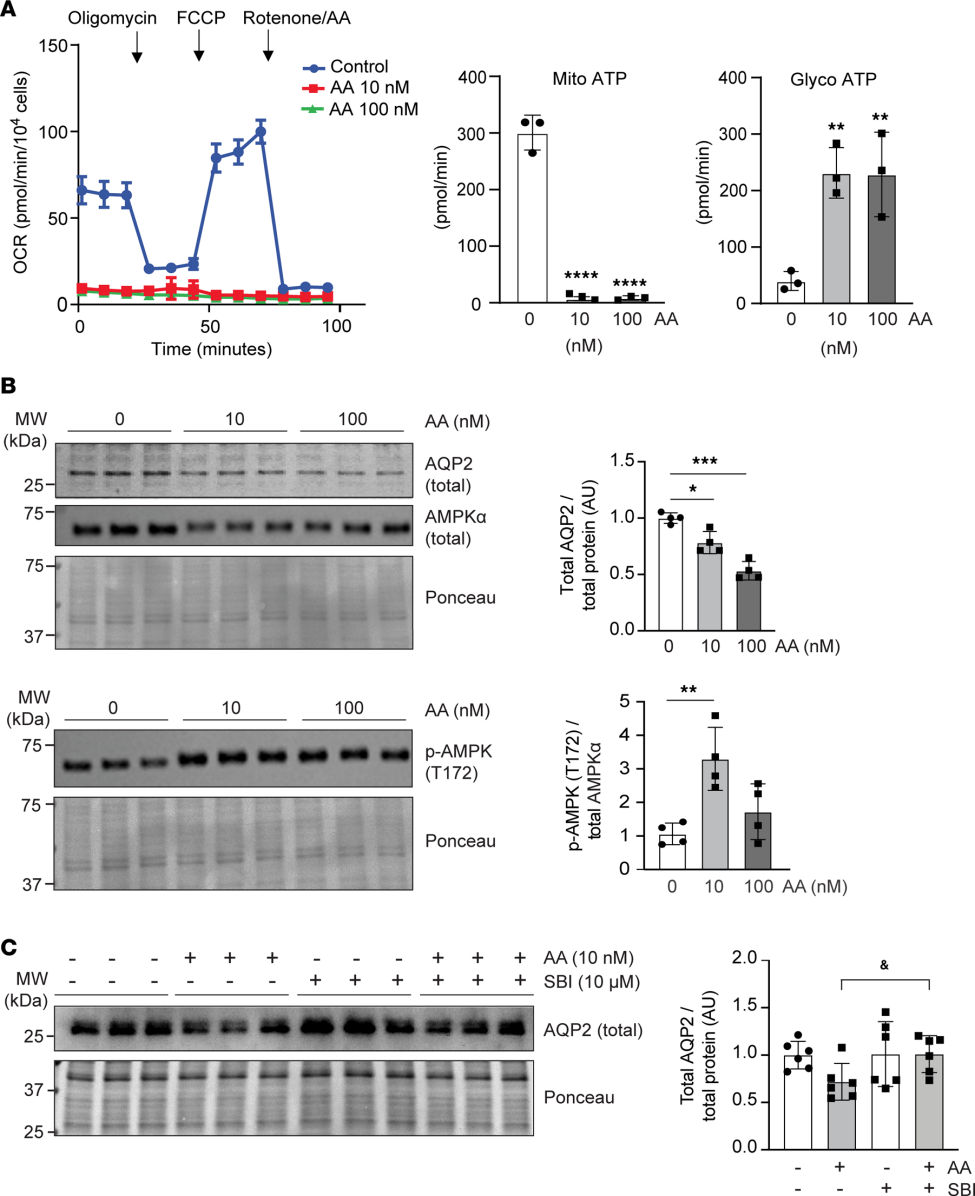
Inhibition of mt complex III decreases AQP2 expression in cultured CD cells. (**A**) Representative oxygen consumption rates (OCRs) in cultured mouse IMCD cells (3 replicates, repeated 3 times) treated with mt complex III inhibitor antimycin A (AA). Shown are the production rates of ATP generated from mitochondrial respiration (Mito ATP) or glycolysis (Glyco ATP) following sequential injections of oligomycin A and rotenone/AA; *n* = 3 for each treatment condition. (**B**) Quantification of total AQP2, total AMPKα, and p-AMPKα (T172) protein levels in mouse IMCD cells treated with AA by immunoblot; *n* = 4, each. (**C**) Quantification of total AQP2 protein levels in AA-treated IMCD cells following addition of AMPK inhibitor SBI-0206965 (SBI) to the culture medium; *n* = 6, each. Data are represented as average mean values ± SD. *P < 0.05; **P < 0.01; **P < 0.001; ***P < 0.001; ****P < 0.0001; ^&^*P* = 0.0324 by 1-way ANOVA with Tukey’s post hoc analysis; for **A**, comparisons were made to control (0 nM). AMPK, 5′ adenosine monophosphate–activated protein kinase; AQP2, aquaporin 2; FCCP, carbonyl cyanide-*p*-trifluoromethoxy phenylhydrazone; IMCD, inner medullary collecting duct; veh, vehicle control; AU, arbitrary units.

To gain insight into the mechanisms by which disruption of mt electron transport affected AQP2 abundance in CD epithelium, we treated cultured inner medullary CD (IMCD) cells with mt complex III inhibitor antimycin A (20). Metabolic flux analysis confirmed that the antimycin A doses used for the experiments had the expected negative effects on mt oxygen consumption rate (OCR) and ATP production (Figure 5A). We found that treatment with antimycin A decreased total AQP2 protein levels in IMCD cells (Figure 5B). This was associated with increased phosphorylation of AMPKα at threonine 172 (T172). To examine the involvement of AMPK in the regulation of total cellular AQP2 abundance, we treated cells with antimycin A in the presence or absence of small molecule AMPK inhibitor SBI-0206965. We found that treatment with SBI-0206965 increased abundance of total cellular AQP2 in IMCD cells and reversed the effects of antimycin A treatment (Figure 5C). This suggested that AMPK activation mediated the effects of pharmacological mt complex III inhibition on AQP2 protein expression in IMCD cells.

**Figure 6:**
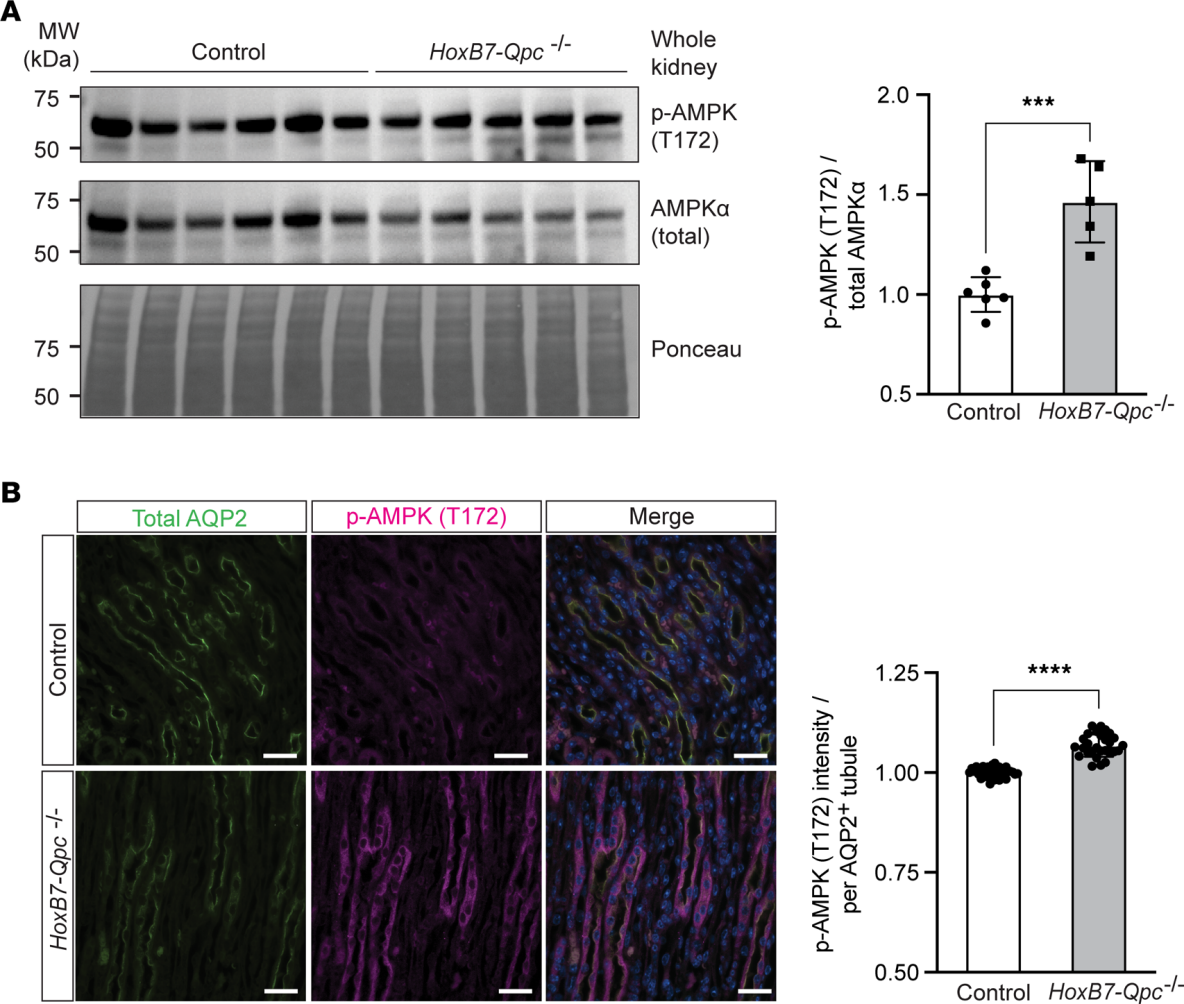
AMPK phosphorylation at threonine 172 is increased in *Qpc^–/–^* CDs. (**A**) Assessment of p-AMPKα (T172) and total AMPKα protein levels in whole-kidney lysates from *HoxB7-Qpc^–/–^* mice by immunoblot. Shown are the relative p-AMPKα (T172) to total AMPKα protein ratios for control (*n* = 6) and *HoxB7-Qpc^–/–^* mice (*n* = 5). (**B**) Representative IF images of medullary CDs stained for AQP2 (green) and p-AMPKα (magenta) in *HoxB7-Qpc^–/–^* kidneys. Nuclei (blue) were stained with DAPI. Shown are the relative normalized IF intensities for p-AMPKα (T172) staining in collecting duct tubules identified by AQP2 expression, *n* = 4 mice per group, 6–8 tubules per mouse. Data are represented as normalized means ± SD. ***P < 0.001, ****P < 0.0001 by 2-tailed Student’s *t* test. Scale bars: 10 μm. AMPK, 5′ adenosine monophosphate–activated protein kinase; AQP2, aquaporin 2.

**Figure 7:**
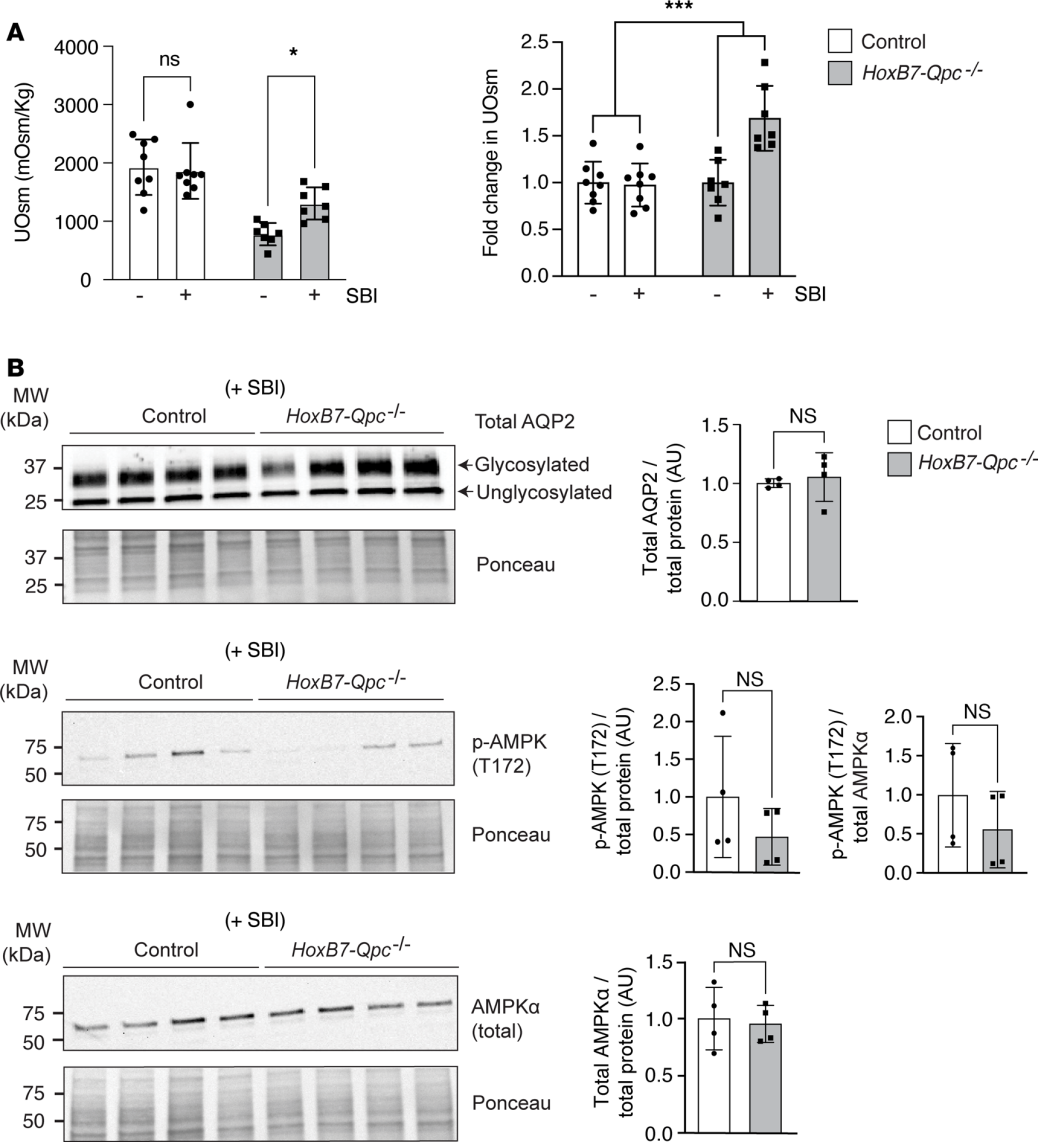
AMPK inhibition increases urine osmolality in *HoxB7-Qpc^–/–^* mice. (**A**) Urine osmolality (UOsm) at baseline and after challenge with AMPK inhibitor SBI-0206965 (SBI) in *Cre^–^* littermate control and *HoxB7-Qpc^–/–^* mice; *n* = 7, each. (**B**) Total AQP2, p-AMPK (T172), and total AMPK protein levels in whole-kidney lysates from *Cre^–^* littermate control and *HoxB7-Qpc^–/–^* mice following treatment with SBI-0206965; *n* = 4, each. Data are represented as average mean values ± SD. *P < 0.01; ***P < 0.001 by 2-way ANOVA followed by Šidák’s multiple-comparison test (**A**) or 2-tailed Student’s *t* test (**B**). NS, not significant. The interaction between genotype and SBI treatment was significant for **A**. AMPK, 5′ adenosine monophosphate–activated protein kinase; AQP2, aquaporin 2; AU, arbitrary units.

To investigate the relationship between AMPK activation and the regulation of AQP2 expression in vivo, we performed immunoblotting of whole-kidney protein extracts and IF staining for activated AMPK. We found that the ratio of p-AMPKα (T172) to total AMPKα protein levels was increased in *HoxB7-Qpc^–/–^* mice compared with *Cre^–^* littermate controls (1.47 ± 0.203 vs. 1.00 ± 0.087). This was associated with a decrease in total AMPKα protein levels in *HoxB7-Qpc^–/–^* kidneys (Figure 6A). In line with these results was the relative IF staining intensity for p-AMPKα (T172), which was increased in AQP2-expressing medullary *Qpc^–/–^* CD cells (Figure 6B). To investigate the effects of AMPK inhibition in vivo, we treated mice with small molecule AMPK inhibitor SBI-0206965 and measured urine osmolality (UOsm) before and 30 minutes after treatment. We found that SBI-0206965 had no marked effect on UOsm in *Cre^–^* littermate control mice (1928 ± 475 vs. 1863 ± 478 mOsm/kg following SBI-0206965 treatment), but substantially increased UOsm in *HoxB7-Qpc^–/–^* mutants (769 ± 191 vs. 1297 ± 275 mOsm/kg) (Figure 7A). Moreover, treatment with SBI-0206965 eliminated the differences in total AQP2, total AMPKα and p-AMPKα (T172) protein levels between control and mutant mice (Figure 7B).

Taken together, our in vitro and in vivo results suggest that AMPK activation is at least partly responsible for the decreased expression of AQP2 in CD cells with mt complex III inactivation, thus providing a biochemical link between mt electron transport and urinary concentration.

## Discussion

In the kidney, transepithelial ion and solute transport is required for the generation and maintenance of osmotic gradients needed for the reabsorption of water through AQP2 water channels. In the current study, we investigated to what degree mt electron transport and oxidative phosphorylation in CD epithelium contributed to the kidney’s ability to concentrate urine. We demonstrate that disruption of mt electron transport resulted in decreased urinary concentrating ability due to AMPK-dependent lower abundance of total cellular AQP2 expression. Our studies identify a mechanistic link between mt electron transport dysfunction, AQP2 regulation, and urinary concentrating ability.

We previously demonstrated that mt complex III was not required for the development of the kidney collecting system (12). One of the principal tasks of the CD is to maintain water homeostasis by concentrating or diluting urine. The ability to concentrate urine necessitates the establishment and maintenance of osmotic gradients for water reabsorption, which is dependent on Na^+^/K^+^-ATPase and the concomitant increase in the expression, activation, and membrane translocation of AQP2 water channels (10). We found that in mice with mt complex III disruption, maximal urine concentration was impaired, while urine was less concentrated under baseline conditions. However, *HoxB7-Qpc^–/–^* mutants were still responsive to water restriction and dDAVP administration, suggesting that in addition to mitochondria-derived ATP, ATP generated by oxygen-independent pathways, such as glycolysis, contributes to osmotic gradient maintenance and water reabsorption in principal CD cells. This notion is consistent with previous reports demonstrating that CD transport processes are supported by glycolysis (21).

Despite the defect in urinary concentration, we did not find differences in baseline serum osmolality or sodium levels between control mice and *HoxB7-Qpc^–/–^* mutants. Although we did not measure oral water intake in our studies, it is likely that *HoxB7-Qpc^–/–^* mutants compensate for increased urinary water loss by increasing their water intake. Since QPC deletion is restricted to kidney CD cells, it is unlikely that abnormal central AVP secretion (i.e., central diabetes insipidus) or primary polydipsia are contributing to the urinary concentration defect observed in *HoxB7-Qpc^–/–^* mutants. This conclusion is supported by the baseline serum chemistries and copeptin levels in mutant mice, which do not indicate any disturbances in central water balance regulation. Whether mt electron transport plays a role in the central regulation of water homeostasis remains an open question and would be an intriguing subject for future investigation.

Our study sheds light on the role of oxidative phosphorylation in CD function. In contrast with proximal tubule epithelial cells, CD cells are characterized by relatively high expression levels of glycolytic enzymes and use mostly glucose as an energy source (2, 22). The observation that mice with defective oxidative phosphorylation are still able to concentrate urine and respond to dDAVP highlights the importance of oxygen-independent ATP generation in CD epithelium. The notion that glycolytic ATP generation is important for CD transport functions is not surprising, as urinary concentration occurs in the renal medulla, which is characterized by the lowest tissue oxygen concentrations in the kidney (7, 8). Nevertheless, further studies are required to generate deeper insights into the effects of specific kidneys stressors, such as fluid restriction and dietary manipulations, on the dynamics of renal tubular energy generation and utilization.

The loss of urinary concentrating ability is an early and universal finding of hypoxic kidney injury (23). In an experimental model of acute kidney injury, Fernandez-Llama and colleagues and Kwon and colleagues found that ischemia, which is associated with mt dysfunction (24, 25), resulted in decreased AQP2 expression and urinary dilution (26, 27). These observations are in line with our studies in which ablation of mt electron transport decreased AQP2 expression and impaired urinary concentration. Additionally, abnormal AQP2 function has been observed in obstructive kidney disease (28), which has been linked to mt oxidative stress (29). Liu et al. suggested that mt oxidative stress secondary to urinary obstruction may play a key role in AQP2 regulation. Specifically, Liu and colleagues reported that treating mice with a cell-permeable superoxide dismutase mimetic (MnTBAP) reversed the obstruction-dependent decrease in the expression of AQP2, but not the expression of AQP1, AQP3, or AQP4 (30). Mt dysfunction has also been associated with the pathogenesis of nephrogenic diabetes insipidus (NDI), which is characterized by polyuria, polydipsia, and low urinary osmolality (31). In its congenital form, NDI is caused by mutations in *AQP2* or *V2R*, and mt damage in CD and other epithelial cells has been detected in kidneys from patients with NDI (32). Taken together with these clinical studies, our data lend support to the notion that mt dysfunction impairs AQP2 function, negatively impacting urinary concentrating ability. To what degree mt dysfunction modulates NDI pathogenesis and severity will have to be investigated in future studies.

Our studies identified AMPK as a negative regulator of AQP2 expression when oxidative phosphorylation is inhibited in CD cells. AMPK is a key modulator of cellular energy homeostasis and a decrease in intracellular ATP levels activates AMPK. Once activated, AMPK inhibits ATP-consuming processes and optimizes cellular energy usage (33, 34). In the kidney, AMPK has been implicated in the regulation of ion channels, pumps, and transporters along the nephron (35). Thus, it is not surprising that during a state of cellular energy depletion, AMPK also affects water clearance via AQP2 water channels. Al-Bataineh et al. demonstrated that acute AMPK activation inhibited AQP2 cell membrane expression in vitro, but did not prevent cell membrane accumulation following stimulation with dDAVP (36). This is in line with our data, as we found that *HoxB7-Qpc^–/–^* mice were still able to respond to dDAVP and that treatment with small molecule AMPK inhibitor SBI-0206965 acutely increased UOsm in *HoxB7-Qpc^–/–^* mice, suggesting a direct effect of AMPK on AQP2. Consistent with Al-Bataineh et al.’s study, we did not find any differences in AQP2 phosphorylation between control and dDAVP-treated mice. However, Al-Bataineh and colleagues reported decreased AQP2 membrane expression in response to AMPK stimulation in vitro, which we did not observe in vivo. Some of the discrepancies between studies could have resulted from differences in experimental design and conditions. Al-Bataineh and colleagues used a direct activator of AMPK, 5-aminoimidazole-4-carboxamide ribonucleoside (AICAR), for a maximum of 4 hours, whereas AMPK activation in our studies was indirect, using antimycin A overnight to inhibit mt complex III. Whether AMPK activation in the setting of intact mt electron transport or the duration of the treatment could have affected membrane expression is unclear. However, our and Al-Bataineh et al.’s study lend strong support to the overarching notion that activation of AMPK impairs AQP2 function.

In contrast with our results, Klein and colleagues treated rats and mice lacking V2R with metformin (AMPK activator) and found increased AQP2 abundance and phosphorylation (37). There are numerous reasons that could account for the differences between what has been reported by Klein et al. and our studies. Metformin was administered in drinking water and thus its effects were not limited to the kidney. Additionally, Klein and colleagues did not probe for p-AMPK (T172) and thus did not assess the activation state of AMPK in kidneys from metformin-treated animals. In contrast, we found that the ratio of p-AMPK (T172) to total AMPK and the abundance of p-AMPK (T172) in the CD was increased in *HoxB7-Qpc^–/–^* mice. The discrepancy between our and Klein and colleagues’ observations could be reconciled by considering that secondary AMPK activation due to genetic mt ETC disruption is likely to result in different effects than the systemic administration of AMPK-activating small molecule compounds. Nevertheless, the observation that metformin increases AQP2 abundance and can do so independently of AVP signaling are compelling (37, 38).

In conclusion, our data demonstrate that disruption of mt electron transport in the renal collecting system impairs maximal concentration of urine due to an AMPK-dependent decrease in the expression of AQP2. Our studies describe a mechanistic link between oxidative phosphorylation and urinary concentrating ability of the kidney and provide rationale for further investigations into the role of mt dysfunction and altered oxygen metabolism in renal water clearance.

## Methods

### Sex as a biological variable.

Both female and male mice were included in the study and analyzed together. Sex was not considered a biological variable.

### Mouse strains and animal procedures.

The generation and genotyping of mice has been described elsewhere (13, 14). All mice received standard 4.5% fat mouse chow ad libitum throughout the studies (5L0D, LabDiet). Ten- to 12-week-old, male and female *HoxB7-cre^tg/+^* *Qpc^fl/fl^* and *Cre^–^* mice were used for the experiments unless otherwise stated. *Cre^–^* littermates were used as control mice. To assess urinary concentration after water restriction, water was withheld for 12 hours overnight and UOsm was measured. To assess maximal urinary concentrating ability over time, mice were injected intraperitoneally (i.p.) with 1 μg/kg of V2R agonist dDAVP (Cayman Chemical), every 12 hours for a total of 6 doses (39–41). To assess the effects of AMPK inhibition in vivo, AMPK antagonist SBI-0206965 (Cayman Chemical) was administered i.p. once, at a dose of 12.5 mg/kg. Spot urine was collected for osmolality measurement 30 minutes after administration of SBI-0206965. Plasma electrolyte concentrations were measured at baseline using a commercially available i-STAT1 blood analyzer and iStat Chem8+ single-use test cartridges (Abbot), for which blood was obtained via cardiac puncture. Copeptin was measured utilizing an ELISA kit for copeptin according to the manufacturer’s instructions (CEA365Mu, Cloud Clone Corporation). UOsm and serum osmolality were measured using a freezing point osmometer from Precision Systems (model 6002).

### DNA and protein analysis.

Genomic DNA analysis was performed as previously described (42). Whole kidneys or cultured cells were lysed in RIPA buffer (Thermo Fisher Scientific) with phosphatase and protease inhibitors (Roche) using a Tissue Tearor homogenizer (model 985370, Daigger Scientific). After lysis, tissues were spun at 21,000*g* and 4°C for 30 minutes. The supernatant was then collected, and total protein quantified by BCA assay (Pierce). Equal amounts of protein were loaded in MiniProtean TGX precast 4%–20% gels (Bio-Rad). Proteins were transferred to nitrocellulose membranes using a Transblot Turbo (Bio-Rad). Total protein was quantified with Ponceau-S (Thermo Fisher Scientific). Membranes were blocked with 5% nonfat dry milk and incubated with primary and secondary antibodies (Supplemental Table 1). Chemiluminescent signal was obtained with Immobilon Western Chemiluminescent HRP (MilliporeSigma) and captured with iBright FL1500 (Thermo Fisher Scientific). Quantification was performed with ImageJ (NIH). For the quantification of total AQP2, bands were combined from both the non-glycosylated (~25 kDa) and the glycosylated AQP2 species (~37 kDa).

### Morphological analysis and imaging.

Kidneys were fixed in 3.7% formaldehyde, 10 mM sodium *m*-periodate, 40 mM phosphate buffer, and 1% acetic acid or in 10% neutral buffered formaldehyde at room temperature overnight (quantification of eGFP^+^ tubules). Fixed tissues were then dehydrated, paraffin-embedded, and sectioned (5 μm) for IF staining. Antibodies for IF studies are listed in Supplemental Table 1. Stained tissues were imaged with a Nikon TiE fully motorized inverted fluorescence microscope, or a Zeiss LSM 980 Confocal AiryScan 2. Slides were scanned with the Aperio VERSA 200 pathology scanning system (Leica Biosystems).

For the quantification of eGFP^+^ tubules per mouse kidney, 5 areas from the cortex (916 × 1651 μm, each) and 3 areas from the medulla (480 × 866 μm, each) were selected from the scanned slides (20× objective) for manual counting. For the quantification of ENaCγ^+^ and AQP2^+^ tubules, slides were scanned utilizing 5×, 20×, and 40× objectives. Data from one additional, “empty” channel were collected concurrently for background and autofluorescent calibrations. Imaging data were stored as.scn files. For image analysis, the original.iff images were extracted from the . file pyramids by individual channel using MatLab (Mathworks). Individual tissue samples located within the whole slide image were identified and extracted. Multiple, random regions were cropped out for machine learning. These images were randomly rotated and reflected to ensure that machine learning was on regions that would not be replicated on the original images. Following supervised machine learning training on these regions, whole tissue images were subsequently processed for feature probability using the software package Ilastik (43). Utilizing MatLab, individual positive tubules were determined with the aid of the Ilastik-generated probability maps. Tubules were then assigned a binary value of “0” if no staining and “1” if staining for ENaCγ or AQP2 was present. The ratio of AQP2^+^ to ENaCγ^+^ tubules was calculated, graphed, and analyzed.

Colocalization IF analysis was performed with NIS-Elements software. Pearson’s correlation was calculated for sections of *Cre^–^* littermate controls and mutant mice for ezrin (Alexa Fluor 647) and total AQP2 (FITC), and p-AQP2 (S269) (Alexa Fluor 555) and total AQP2 (FITC) (*n* = 4 per group). Two images were taken per mouse and at least 5 tubules per image were selected. Pearson’s correlation analysis was performed for each tubule. Individual values for each tubule were graphed and analyzed with a Student’s *t* test. Fluorescence intensity was assessed with NIS-Elements software. AQP2^+^ tubules were selected, and mean fluorescence intensity was measured for p-AMPKα (T172) (Alexa Fluor 647). Two images were acquired per mouse and at least 5 tubules were selected per image (*n*= 4 per group). Normalized intensity was then plotted and analyzed with a Student’s *t* test. Images analysis was performed in a blinded fashion.

### Cell culture.

We used C57BL/6J immortalized IMCD cells for all in vitro experiments (44). Cells were grown in low-glucose DMEM (Gibco/Thermo Fisher Scientific) supplemented with 10% fetal bovine serum (Gibco/Thermo Fisher Scientific), 100 IU/mL penicillin, and 100 μg/mL streptomycin (Gibco/Thermo Fisher Scientific). Cells were plated in 12-well dishes at a density of approximately 5 × 10^5^ cells per well and grown to confluence over 36–48 hours. Twenty-four hours prior to the experiment, cell medium was changed to serum-free DMEM. Antimycin A (Cayman Chemical) and/or 10 μM SBI-0206965 (Cayman Chemical) or vehicle was added to cell culture wells at this time point.

### Metabolic flux analysis.

IMCD cells were seeded into microplates at a density of 20,000 cells/well. Mito Stress Test kits (Agilent Technologies) and ATP Rate Assay kits (Agilent Technologies) were used to measure OCR and ATP production with the Agilent Seahorse XFe24 metabolic flux analyzer, according to the manufacturer’s instructions. Data were normalized to cell number as determined by nuclear staining using the BioTek Cytation 5 image reader.

### Statistics.

Data are reported as mean ± SD. Statistical analyses were performed with Prism 10 software (GraphPad Software Inc.), using a 2-tailed Student’s *t* test, 1-way ANOVA with a post hoc Tukey’s test, or 2-way ANOVA with a post hoc Šidák’s test, as indicated. *P* values of less than 0.05 were considered statistically significant.

### Study approval.

All procedures involving mice were performed in accordance with the NIH *Guide for the Care and Use of Laboratory Animals* (National Academies Press, 2011) and were reviewed and approved by the Institutional Animal Care and Use Committee (IACUC) of Vanderbilt University, Nashville, Tennessee, USA.

### Data availability.

Raw data are available in the Supporting Data Values file or directly from the corresponding author upon reasonable written request.

## Author contributions

JPA and VHH conceived the study and designed experiments. JSC, RB, YZ, JBT, FB, OD, HK, and JTR performed experiments. JSC, YZ, JPA, AST, FB, JAW, RB, and VHH analyzed data. JC, RB, JPA, and VHH wrote and revised the manuscript, and made figures. JAW, FB, and AST edited the manuscript.

## Supplementary Material

Supplemental data

Unedited blot and gel images

Supporting data values

## Version 1 10/03/2024

In-Press Preview

## Version 2 11/22/2024

Electronic publication
