# Supplementary material for: Disruption of mitochondrial electron transport impairs urinary concentration via AMPK-dependent suppression of aquaporin 2
Source: JCI Insight. 2024 Nov 22;9(22):e182087. doi: 10.1172/jci.insight.182087 (PMC11601893; doi:10.1172/jci.insight.182087)
Supplement: Supplemental data [file jciinsight-9-182087-s119.pdf]

SUPPLEMENTARY MATERIALS

Disruption of mitochondrial electron transport impairs urinary concentration via AMPK-dependent suppression of aquaporin-2

Joshua S. Carty, Ryoichi Bessho, Yvonne Zuchowski, Jonathan B. Trapani,
Olena Davidoff, Hanako Kobayashi, Joseph T. Roland, Jason A. Watts, Andrew S. Terker, Fabian Bock,
Juan Pablo Arroyo and Volker H. Haase

Table of Contents:

Supplementary Table S1
Supplementary Table S2
Supplementary Figure S1
Supplementary Figure S2
Supplementary Figure S3
Supplementary Figure S4

Supplementary Table S1

Primary and secondary antibodies used for immunofluorescence and immunoblotting.

Antibody / type	Host	Catalog Nr.	Vendor	Dilution	Time
AMPKα (D5A2)	rabbit	5831	Cell Signaling Technology, Danvers, MA, USA	WB 1:1000<br>IF 1:250	2 hr RT
p-AMPKα (T172) (40H9)	rabbit	2535	Cell Signaling Technology, Danvers, MA, USA	WB 1:1000<br>IF 1:250	3 hr RT
AQP2 (total) - HRP conjugated	mouse	sc-515770	Santa Cruz Biotechnology, Dallas, TX, USA	IF 1:100,<br>WB 1:200	o/n, 4^0C
AQP2 (total) - FITC conjugated	mouse	sc-515770	Santa Cruz Biotechnology, Dallas, TX, USA	IF 1:100,<br>WB 1:500	1 hr RT
AQP2 (total) - AF647 conjugated	mouse	sc-515770	Santa Cruz Biotechnology, Dallas, TX, USA	IF 1:100,<br>WB 1:500	1 hr RT
AQP2 (total) - non-conjugated	mouse	sc-515770	Santa Cruz Biotechnology, Dallas, TX, USA	IF 1:100	o/n 4^0C
p-AQP2 (S269)	rabbit	p112-269t	PhosphoSolutions, Aurora, CO, USA	WB 1:1000<br>IF 1:250	1 hr RT
AQP3 (total)	Rabbit	HPA014924	Millipore Sigma, Burlington, MA	WB 1:1000	o/n 4^0C
ENaCγ	rabbit	SPC-405D	StressMarq Bioscience, Victoria, BC, Canada	IF 1:250,<br>WB 1:1000	1 hr RT
Ezrin	rabbit	3145	Cell Signaling Technology, Danvers, MA, USA	IF 1:500	1 hr RT
NA^+/K^+ ATPase α1/ATP1A1 - AF546 conjugated	mouse	sc-514614	Santa Cruz Biotech, Dallas, TX, USA	WB 1:500	1 hr RT
GFP	rabbit	ab13970	Abcam, Waltham, MA, USA	IF 1:200	o/n, 4^0C
tdTomato / RFP	rabbit	600-401-379	Rockland Immunochemicals, Limerick, PA, USA	IF 1:200	o/n, 4^0C
anti-rabbit IgG - HRP conjugated	Goat	111-035-003	Jackson Immunoresearch, West Grove, PA, USA	WB 1:5000	1 hr RT
anti-rabbit IgG AF 647 conjugated	Donkey	711-605-152	Jackson Immunoresearch, West Grove, PA, USA	WB 1:5000	1 hr RT
anti-rabbit IgG (H+L)-Cy3	Donkey	711-166-152	Jackson Immunoresearch, West Grove, PA, USA	IF 1:200	1 hr RT
anti-mouse IgG (H+L)-Cy5	Donkey	715-175-150	Jackson Immunoresearch, West Grove, PA, USA	IF 1:200	1 hr RT
anti-Chicken IgY (H+L) Alexa Fluor 488	Goat	A-11039	Thermo Fisher Scientific, Waltham, MA, USA	IF 1:500	1 hr RT

Abb.: IF, immunofluorescence; o/n, overnight; RT, room temperature; WB, Western blot.

Supplementary Table S2

Serum electrolytes in control and *HoxB7-Qpc*$^{-/-}$ mice.

Genotype	nbr	Na^+ (mmol/L)	K^+ (mmol/L)	Cl^- (mmol/L)	iCa^{2+} (mg/dL)	HCO_3^- (mmol/L)	Anion gap
Control	n=3	147.7 ± 0.5	3.2 ± 0.2	107.3 ± 1.2	1.30 ± 0.03	24.3 ± 0.5	20.0 ± 0.8
HoxB7-Qpc$^{-/-}$	n=3	146.7 ± 0.9	3.5 ± 0.5	109.0 ± 1.6	1.30 ± 0.01	22.3 ± 2.1	19.7 ± 0.5

Abb.: nbr, number of mice.

Supplementary Figures

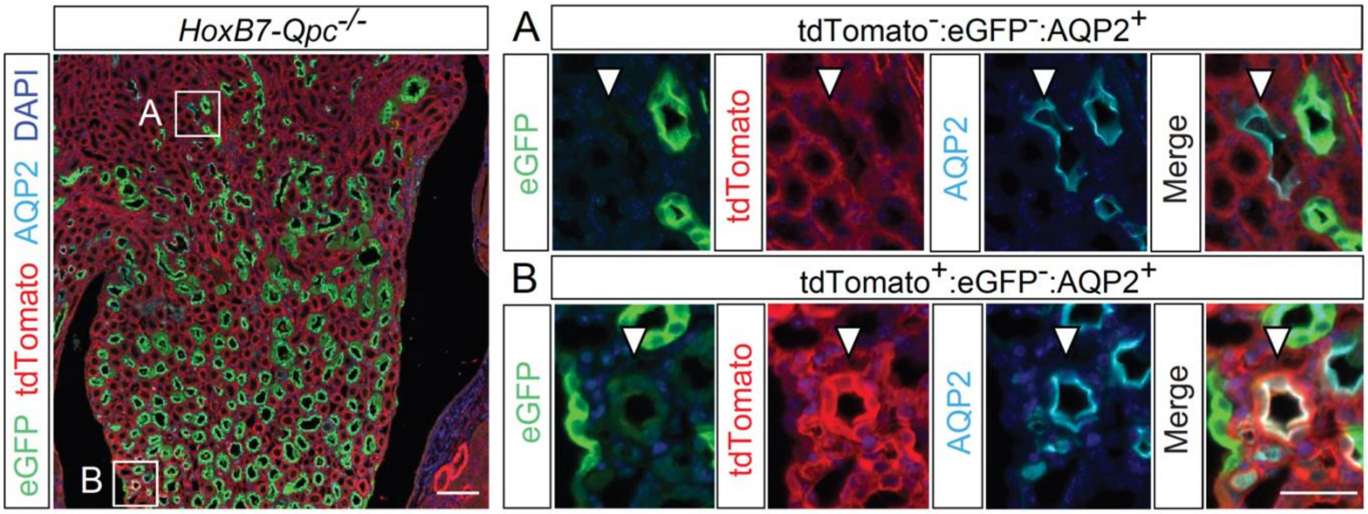

Supplementary Figure S1. Efficient recombination of *Qpc* in *HoxB7-Qpc*$^{-/-}$ kidneys (linked to Figure 1). To investigate whether AQP2(+):eGFP(-) epithelial cells represent CD cells that did not recombine or cells with an inactive Cre-reporter allele, we performed co-staining for tdTomato (tdT) in 6-month-old *HoxB7-Qpc*$^{-/-}$ mice. At baseline (no recombination), the active reporter allele expresses tdT (red fluorescence). Cre-mediated recombination switches the expression from tdT to eGFP. The lack of tdT expression in eGFP(-):AQP2(+) cells (panel A) indicates that the reporter is not active, whereas tdT expression in eGFP(-):AQP2(+) cells (panel B) indicates that Cre-mediated recombination did not occur. Scale bars: 100 µm (low magnification), 50 µm (high magnification). Abb.: AQP2, aquaporin 2; DAPI, 4',6-diamidino-2-phenylindole; eGFP, enhanced green fluorescence protein.

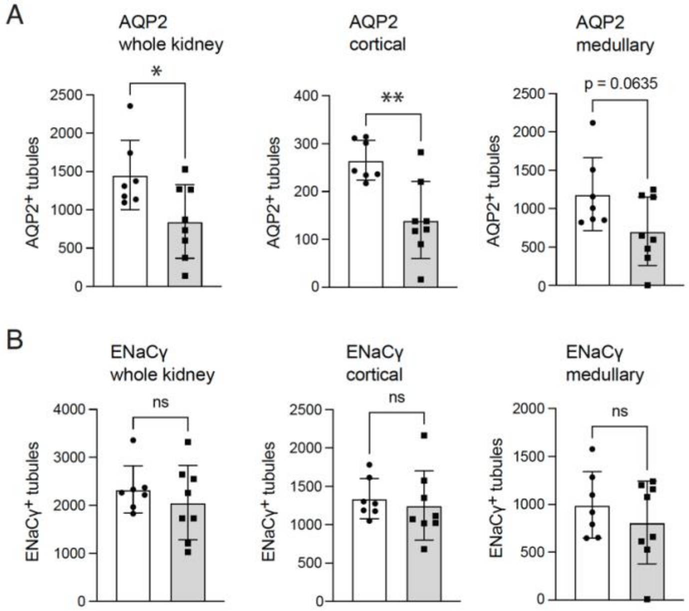

Supplementary Figure S2. AQP2-expressing tubules are reduced in *HoxB7-Qpc*$^{-/-}$ kidneys (linked to Figure 2**)**. (A) Counts of AQP2(+) tubules, and (B) ENaCγ(+) tubules in cortex, medulla, or whole kidney. For each kidney section, all tubules were counted. Counts were then segregated by region; *Cre(-)* littermate control (n=7) and *HoxB7-Qpc*$^{-/-}$ mice (n=8); *Cre(-)* littermate control (n=7) and *HoxB7-Qpc*$^{-/-}$ mice (n=8). Data are represented as average mean values ± SD; two-tailed Student's *t*-test, *$P < 0.05$, **$P < 0.01$; ns = not significant.

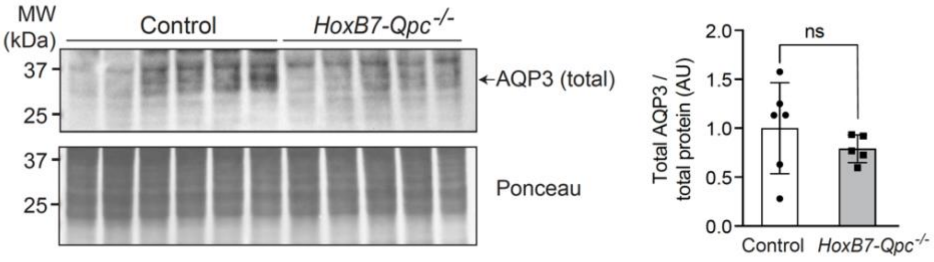

Supplementary Figure S3. **Total AQP3 protein abundance in *HoxB7-Qpc*$^{-/-}$ mice** (linked to Figure 3). Total aquaporin-3 (AQP3) protein levels in whole kidney lysates from *Cre(-)* littermate control and *HoxB7-Qpc*$^{-/-}$ mice; n = 6 and 5, respectively. Data are represented by mean values ± SD; two-tailed Student's *t*-test, ns = not significant. Abb.: AU, arbitrary units.

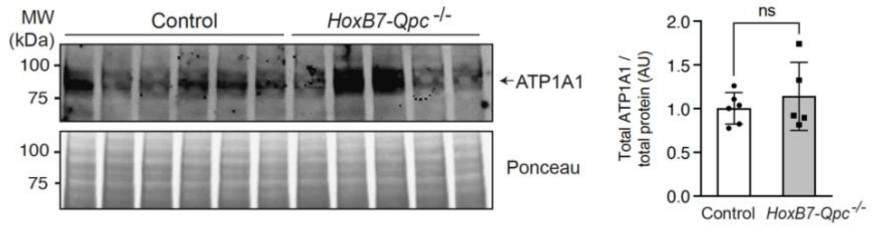

Supplementary Figure S4. **Total Na^{+}/K^{+} ATPase protein abundance in *HoxB7-Qpc*$^{-/-}$ mice** (linked to Figure 3). Total Na^{+}/K^{+} ATPase α1 (ATP1A1) protein levels in whole kidney lysates from *Cre(-)* littermate control and *HoxB7-Qpc*$^{-/-}$ mice; n = 6 and 5, respectively. Data are represented by mean values ± SD; two-tailed Student's *t*-test, ns = not significant. Abb.: AU, arbitrary units.
