# Supplementary material for: Disruption of mitochondrial electron transport impairs urinary concentration via AMPK-dependent suppression of aquaporin 2
Source: JCI Insight. 2024 Nov 22;9(22):e182087. doi: 10.1172/jci.insight.182087 (PMC11601893; doi:10.1172/jci.insight.182087)

Figure 1 - DNA gel to assess *Qpc* recombination by genomic PCR

Figure 1A

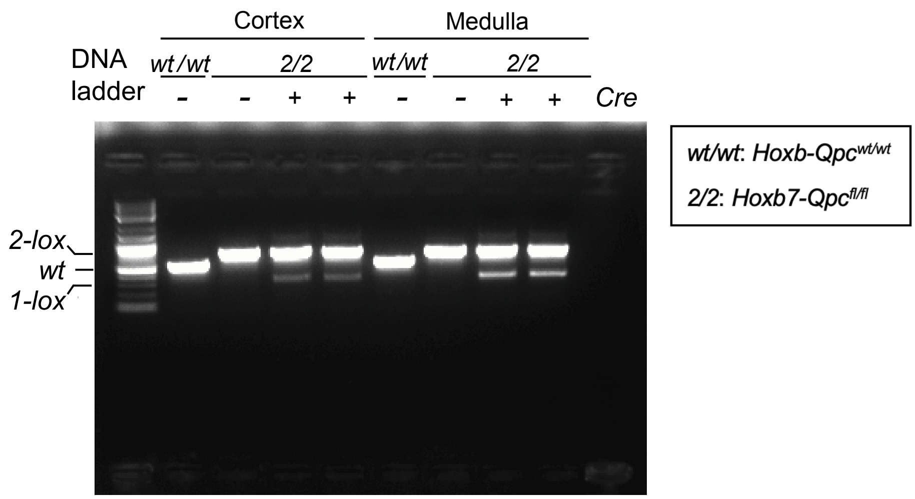

Figure 3 - Whole kidney lysates used to assess total AQP2

Figure 3A

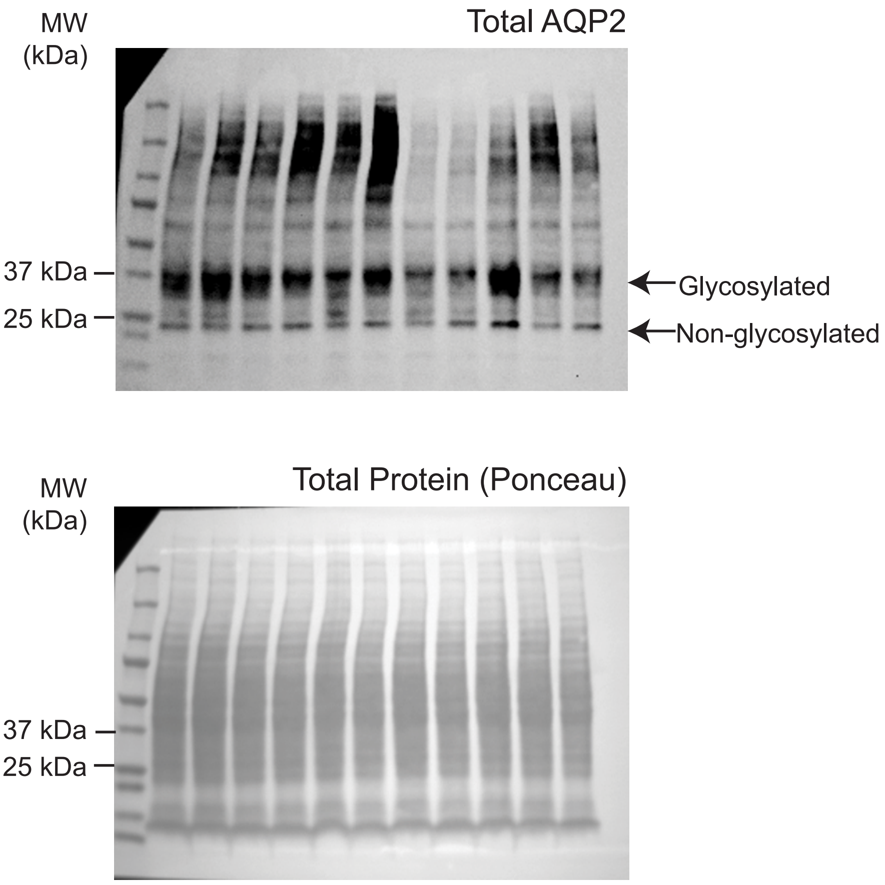

UNCUT GELS 2

Figure 5 - IMCD cell lysates treated with antimycin A and/or SBI

Figure 5B, Panel 1

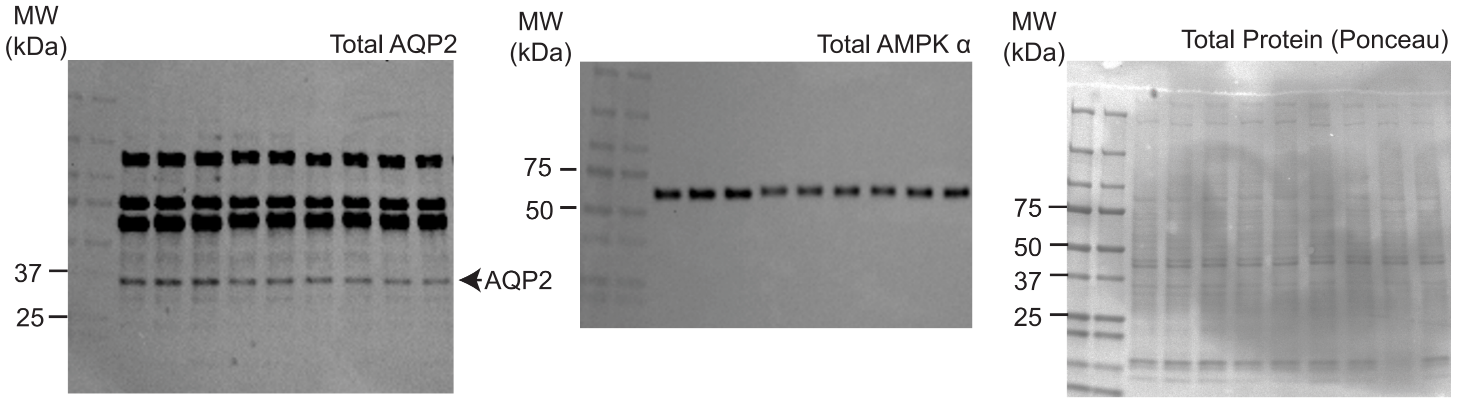

Figure 5B, Panel 2

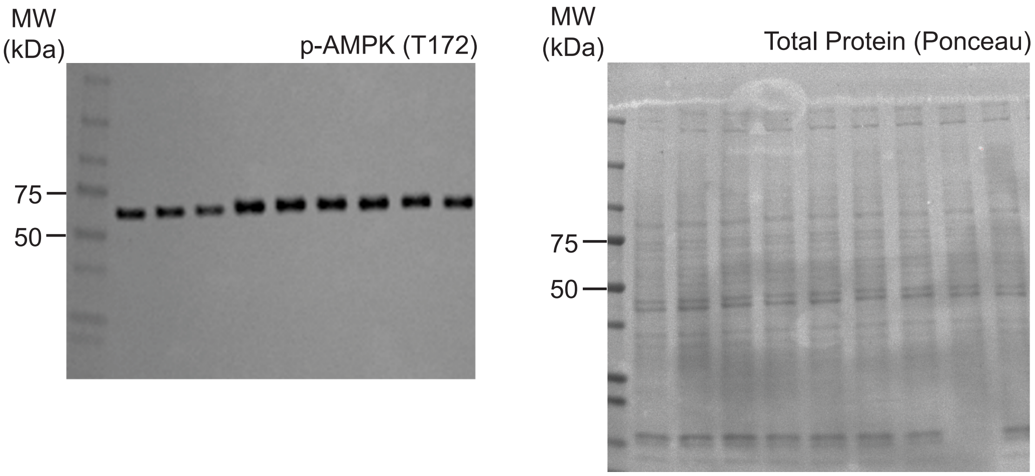

Figure 5C

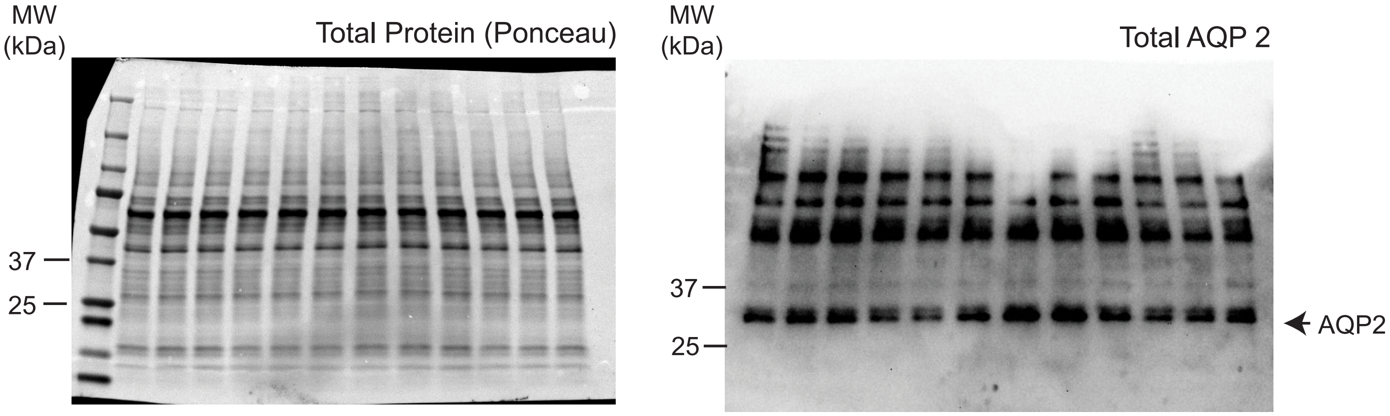

Figure 6 - Whole kidney lysates used to assess total AMPKα and p-AMPK (T172)

Figure 6A

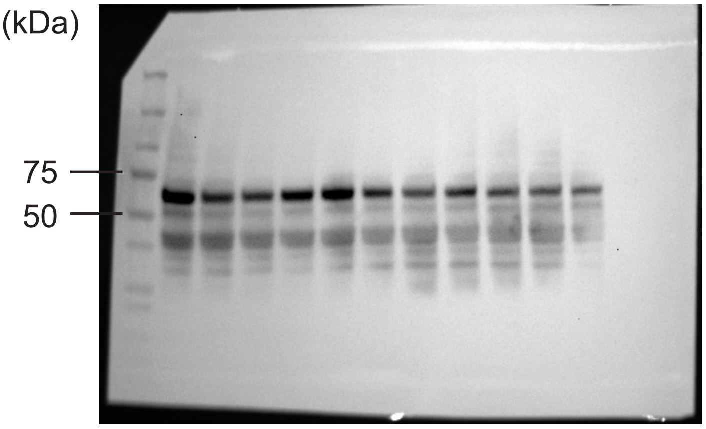

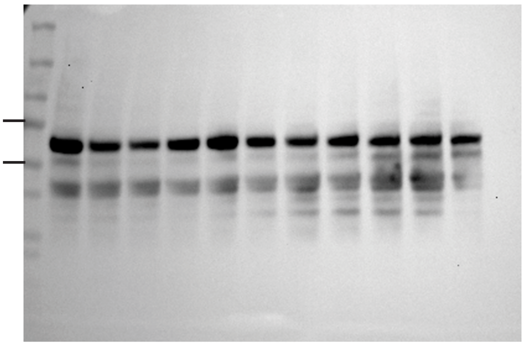

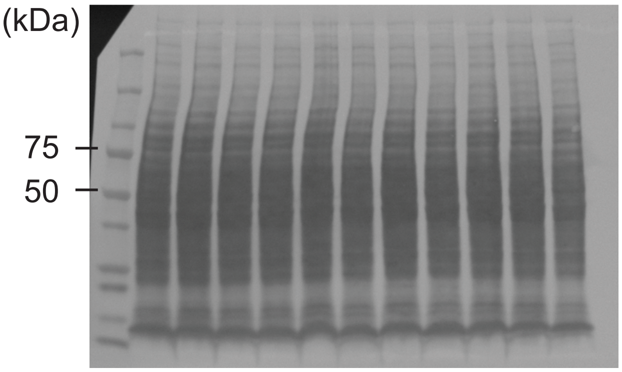

Figure 7 - Whole kidney lysates used to assess total Aqp2, AMPK, and p-AMPK (T172) post SBI treatment

Figure 7B

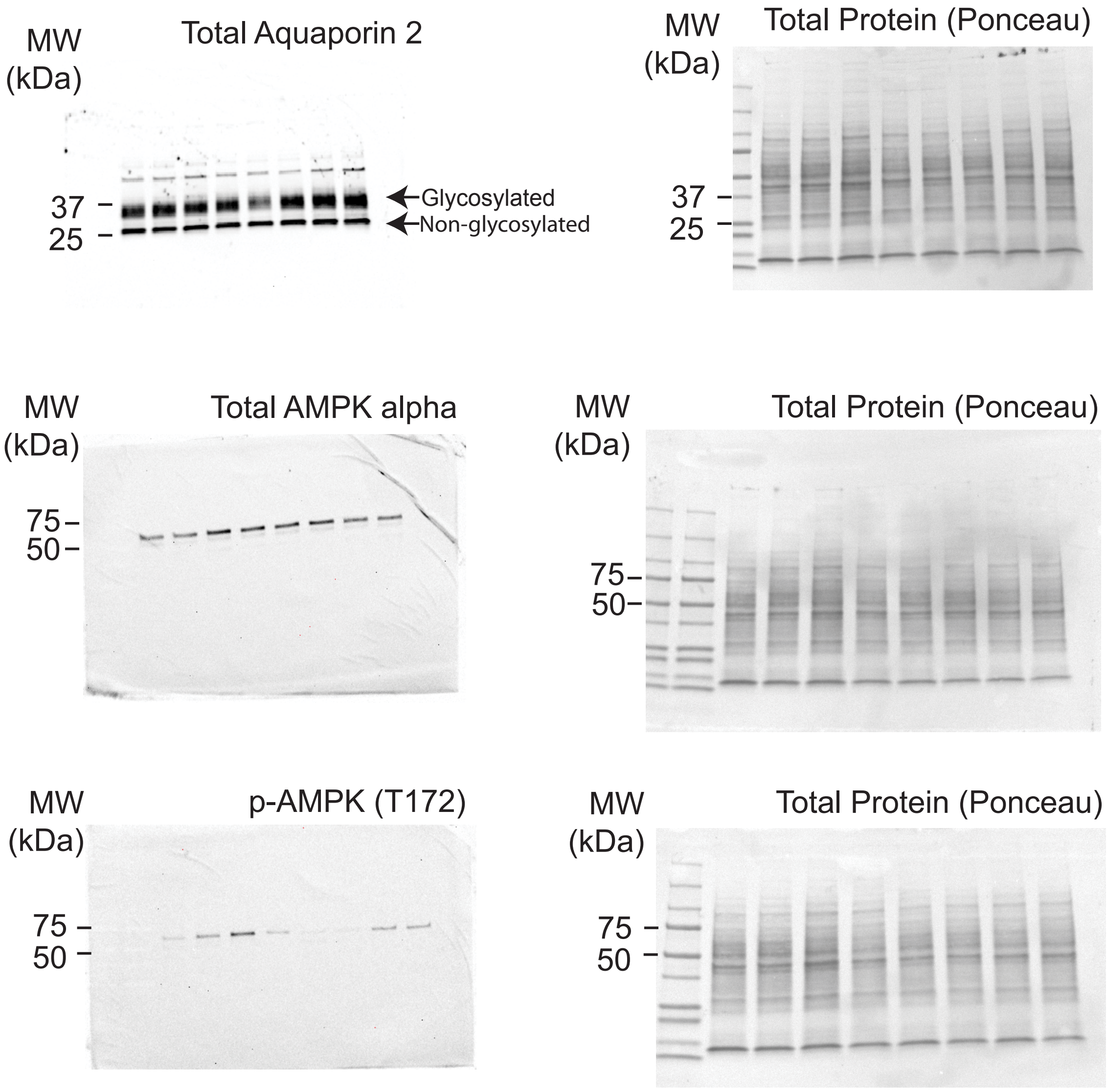

UNCUT GELS 5

Supplementary Figure S3 - Whole kidney lysates used to assess total AQP3

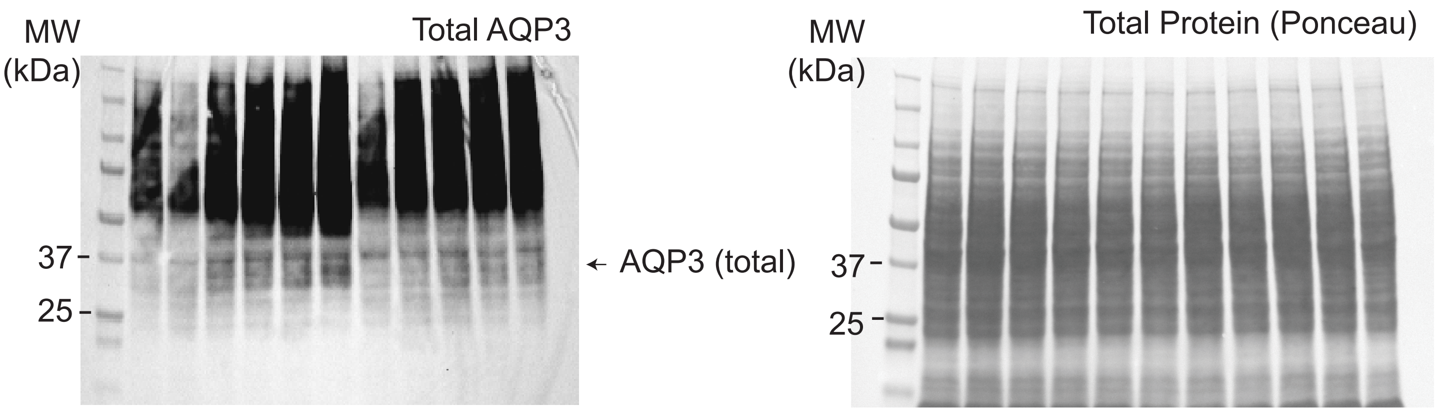

Supplementary Figure S4 - Whole kidney lysates used to assess total Na^+/K^+ ATPase α1

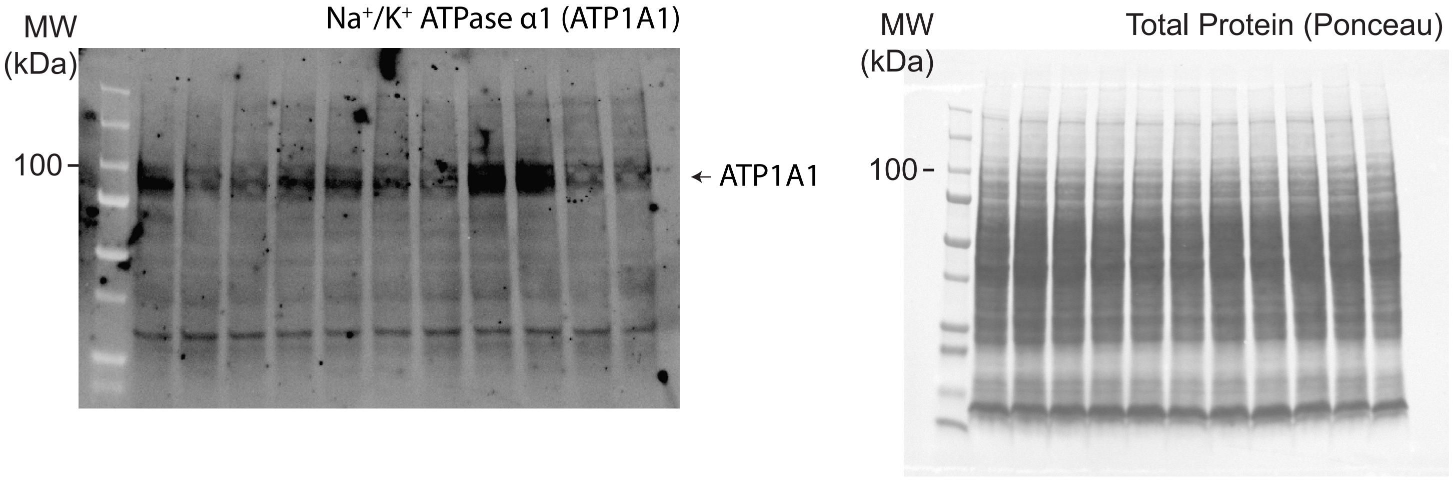

Supplement: Unedited blot and gel images [file jciinsight-9-182087-s120.pdf]
